# Chronic Post-craniotomy Fistula Persisting for Seven Years in a Patient With Neurofibromatosis Type 2

**DOI:** 10.7759/cureus.107685

**Published:** 2026-04-25

**Authors:** Julia L Armstrong, Oren G Nedjar, Amanda Morello, Prakriti Singh Shrestha, Diego Muritiba, Inima Rodriguez Caballero

**Affiliations:** 1 Medicine, Dr. Kiran C. Patel College of Osteopathic Medicine, Nova Southeastern University, Fort Lauderdale, USA; 2 Family Medicine, Larkin Community Hospital, Miami, USA

**Keywords:** draining fistula, intracranial osteomyelitis, neurofibromatosis type 1 or type 2, neurofibromatosis type 2, post-craniotomy

## Abstract

Chronic post-craniotomy draining fistulas are rare complications, particularly in patients who have received prior antibiotic therapy. Individuals with neurofibromatosis type 2 (NF2) may face heightened risk due to repeated cranial interventions, impaired wound healing, radiation exposure, and tumor burden. Persistent fistulas in this population are infrequently reported. We describe an unusual case of a chronic post-craniotomy draining fistula persisting for more than seven years in a patient with NF2 who had undergone multiple cranial surgeries and prior radiation therapy for tumor management, along with an antibiotic course for osteomyelitis in the past. Despite intermittent antibiotic treatment and conservative wound care, the draining sinus tract remained unresolved. This case underscores the importance of maintaining a high index of suspicion for chronic fistula formation in complex neurosurgical patients, even years after the index procedure. Early recognition and multidisciplinary management are essential to prevent prolonged morbidity and optimize surgical outcomes.

## Introduction

A persistent draining fistula following craniotomy, in the absence of cerebrospinal fluid (CSF) leakage or abnormal vascular shunting, represents a rare post-operative complication [1]. Reported etiologies include retained foreign bodies, chronic infection, frontal sinus violation, osteomyelitis, and biofilm formation along implanted materials [1]. Patients may present with headache, persistent purulent drainage, localized scalp swelling, or intermittent wound breakdown [1].

Prolonged draining fistulas over several years are rarely described in the literature, with most reported cases resolving within months. Patients with neurofibromatosis type 2 (NF2) represent a unique population that may be at increased risk for post-operative complications given repeated cranial surgeries, radiation exposure, and altered tissue integrity [2]. NF2 is characterized by the development of multiple intracranial and spinal tumors, including vestibular schwannomas, ependymomas, and meningiomas [2]. The cumulative effects of repeated surgical dissection, prior radiation therapy, and tumor-related mass effect may predispose these patients to impaired wound healing and chronic inflammatory states [3].

Computed tomography (CT) remains the imaging modality of choice in the initial evaluation, as it allows assessment of bony involvement, hardware complications, and sinus tract formation [4]. Magnetic resonance imaging (MRI) may provide further delineation of soft tissue and intracranial pathology when indicated [4].

The management of post-craniotomy surgical site infections involves integrated medical and surgical approaches. Available literature supports the use of topical vancomycin and multimodal peri-operative prevention bundles that combine antiseptic preparations, time-sensitive antibiotic use, precise operative techniques, and strict post-operative wound care [5]. A chronic post-craniotomy fistula often requires more than local debridement, as infection within the sinus outflow tract can serve as a continuous source of recurrent infection.

A more durable management through cranialization techniques, involving debridement of diseased tissue and separation of the sinonasal and intracranial spaces, has shown more definitive resolution of infectious processes and fistula formation [4]. But in our patient, given the absence of intracranial involvement and the superficiality of the infection, the chronic fistula was managed with a more conservative approach, which incorporated antibiotic therapy and drainage; however, the extended duration of the fistula likely limited tissue healing and contributed to the recurrence of the infectious process and chronic drainage of the tract despite non-operative measures.

We present a rare case of a chronic post-craniotomy draining fistula that has persisted for over seven years in a patient with NF2, highlighting potential mechanisms and risk factors contributing to prolonged sinus tract formation despite antibiotic therapy.

## Case presentation

A 59-year-old woman with a history of neurofibromatosis type 2 (NF2), recurrent meningiomas status post-multiple surgical interventions complicated by resolved osteomyelitis, Parkinson’s disease, seizure disorder, and hyperlipidemia presented with persistent purulent drainage from a chronic fistula at a prior craniotomy site. The patient was not immunocompromised. Her home medications included atorvastatin 20 mg nightly, carbidopa-levodopa 25/100 mg three times daily, and temazepam 30 mg at bedtime.

She initially underwent a left frontal craniotomy in 2013 in Havana, Cuba, for meningioma resection, complicated by post-operative infection requiring bone-flap removal. After establishing care in the United States in 2017, residual meningioma was treated with gamma knife radiosurgery (November 15, 2017). She subsequently underwent cranioplasty on March 30, 2018, and received a second gamma knife treatment (September 24, 2019) for interval tumor growth superior to the original surgical site.

In February 2023, she developed wound dehiscence and underwent irrigation and debridement with primary closure; cultures were negative. Despite this intervention and multiple antibiotic courses, including one month of doxycycline and levofloxacin on July 1, 2025, she continued to have persistent scant drainage.

On October 9, 2025, she presented again with seven days of increased purulent discharge. She denied fever, headache, photophobia, neck stiffness, nausea, vomiting, or focal neurological deficits. Vital signs were stable. Physical examination revealed a chronic wound in the left temporal region without surrounding erythema, edema, or cellulitis. Laboratory evaluation showed no leukocytosis (WBC: 7-9×10⁹/L), low-to-normal C-reactive protein (<0.5 mg/dL), and mildly elevated erythrocyte sedimentation rate (24-34 mm/h) (Table 1).

**Table 1:** Admission laboratory values.

Parameters	Values	Reference range
White blood cell count (WBC)	7-9×10⁹/L	4-11×10⁹/L
C-reactive protein (CRP)	<0.5 mg/dL	<1.0 mg/dL
Erythrocyte sedimentation rate (ESR)	24-34 mm/h	0-20 mm/h

**Figure 1:**
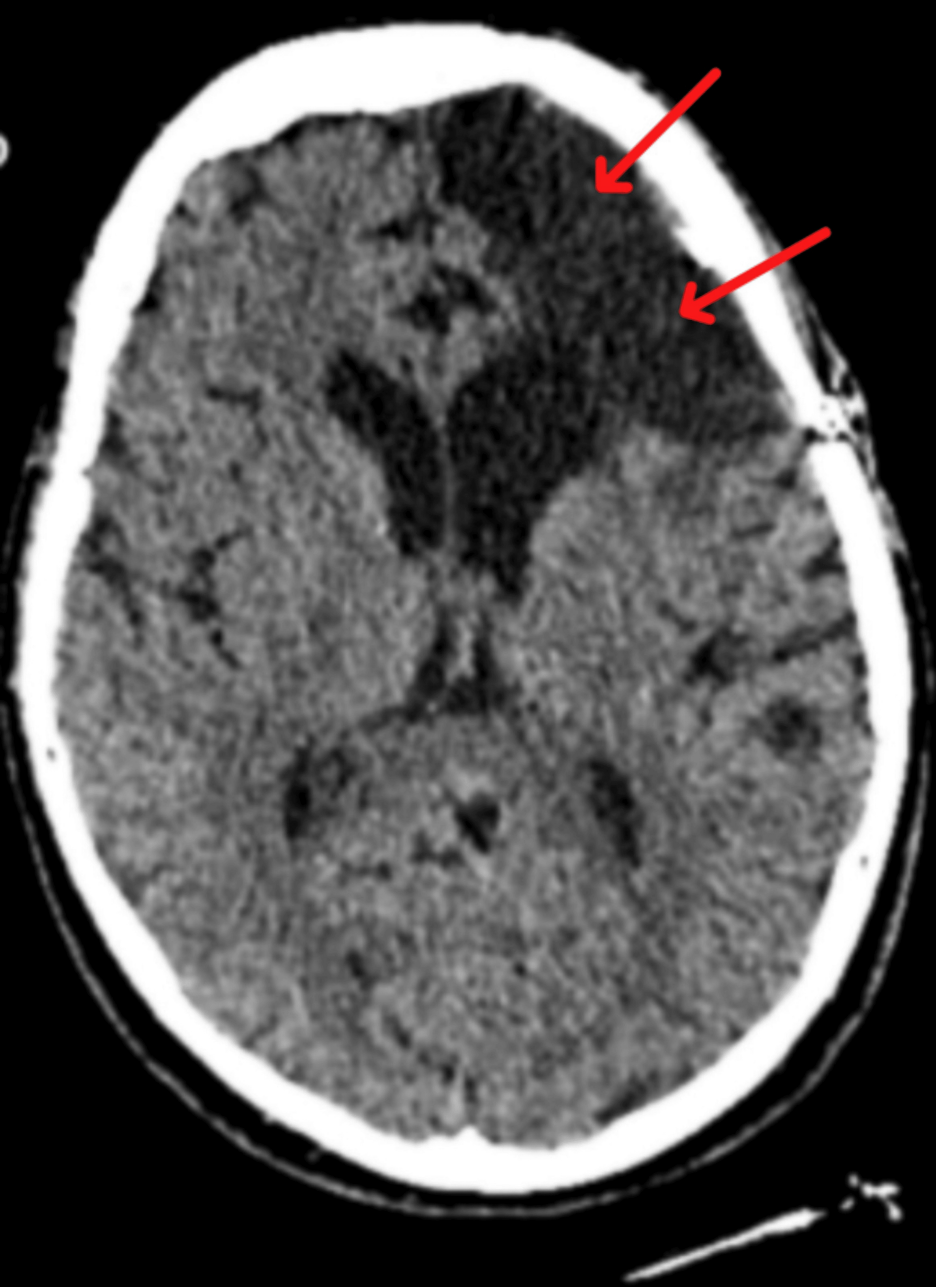
Non-contrast CT of the brain. Image demonstrating prior left frontal craniotomy changes. Red arrows indicate areas of hypodensity and volume loss consistent with post-operative encephalomalacia at the surgical margins, without evidence of acute intracranial infection.

Computed tomography of the brain without contrast demonstrated prior left frontal surgical changes and residual meningiomas at the superior and inferior margins of the craniotomy site, without radiographic evidence of acute intracranial infection (Figure 1).

Empiric intravenous vancomycin was initiated pending wound and blood culture results. Methicillin-resistant *Staphylococcus aureus* (MRSA) screening was negative; however, wound cultures grew methicillin-sensitive *Staphylococcus aureus* (MSSA). Following consultation for infectious disease, vancomycin was discontinued, and therapy was transitioned to cefepime based on susceptibilities.

During a three-day hospitalization, the patient remained afebrile and hemodynamically stable without clinical or laboratory evidence of systemic infection. Drainage from the craniotomy site decreased, and no new neurological deficits were observed. Given the absence of intracranial involvement or deep tissue extension on imaging, neurosurgery and infectious disease recommended conservative outpatient management without immediate surgical intervention.

She was discharged on oral linezolid 600 mg twice daily and ciprofloxacin 500 mg twice daily for 10 days, selected for broad antimicrobial coverage, favorable bone penetration, and high oral bioavailability. Close outpatient follow-up was arranged with the infectious disease in two weeks and the neurosurgery in four weeks. The patient’s complete neurological and surgical timeline is summarized in Table 2.

**Table 2:** Clinical timeline of patient course. MSSA: methicillin-sensitive *Staphylococcus aureus*

Timeline	Event	Description
2013	Initial craniotomy	Craniotomy performed in Havana, Cuba; post-operative infection led to bone-flap removal.
2017	Care established	Patient established care with the doctor; residual meningioma identified and treated with gamma knife radiosurgery.
November 15, 2017	Gamma knife radiosurgery	Gamma knife performed for residual meningioma.
March 30, 2018	Cranioplasty	Cranioplasty was performed to reconstruct the skull defect.
September 24, 2019	Further radiosurgery	An additional tumor superior to the craniotomy site was treated with the gamma knife.
February 9, 2023	Wound dehiscence	Wound dehiscence was managed with incision and drainage and closure; intermittent clear drainage persisted.
July 1, 2025	Chronic fistula re-evaluation	Chronic fistula with purulent drainage re-evaluated; oral antibiotics given, but drainage persisted.
October 9, 2025	Hospital admission	Admitted for increased purulent drainage; imaging showed no acute infection; labs mostly normal.
October 10, 2025	Antibiotic adjustment	Wound culture positive for MSSA; vancomycin discontinued, cefepime initiated based on sensitivities.
October 12, 2025	Discharge and plan	Discharged stable; chronic fistula persists; outpatient antibiotics and follow-up recommended; no surgery planned.

## Discussion

Chronic post-craniotomy draining fistulas are uncommon but clinically significant complications, most often reflecting an underlying structural or infectious process [1]. In this patient, the persistence of external drainage for more than seven years strongly suggests biofilm-associated infection involving retained cranioplasty material. Prosthetic implants can serve as a nidus for bacterial adhesion and biofilm formation, enabling organisms to evade host immune defenses and develop relative antibiotic tolerance [6]. Once established, biofilms create a protected microenvironment that limits antimicrobial penetration and promotes chronic, smoldering infection, frequently necessitating surgical debridement or hardware removal for definitive resolution [6,7].

Microbiology

Microbiologically, cultures grew methicillin-sensitive *Staphylococcus aureus* (MSSA), a well-recognized pathogen in post-operative neurosurgical infections [8]. Empiric broad-spectrum therapy was narrowed once susceptibilities were available, and oral agents with favorable bone penetration and high bioavailability were selected to facilitate outpatient management [9,10]. Despite appropriate antimicrobial coverage, the prolonged course underscores the inherent difficulty in eradicating implant-associated infections without addressing the underlying structural substrate.

Patient risk factors

This patient had significant predisposing factors, including prior craniotomy with bone-flap removal (2013), cranioplasty (2018), and multiple stereotactic radiosurgeries, including gamma knife (2017, 2019). Repeated surgical interventions disrupt scalp vascularity, while radiation induces microvascular injury, fibrosis, and chronic inflammation, collectively impairing wound healing and tissue perfusion, which ultimately limit effective immune response and antibiotic delivery [11,12]. In patients with neurofibromatosis type 2, tumor burden and associated vasculopathy may further compromise microvascular integrity, increasing susceptibility to delayed wound breakdown and chronic sinus tract formation [13].

Decision-making: conservative management vs. surgical explantation

The decision between conservative management and surgical explantation in post-cranioplasty infections must be individualized. Factors favoring conservative therapy include clinical stability, absence of intracranial extension, and superficial infection responsive to antibiotics. Conversely, deep infection, persistent drainage, hardware involvement, or failure of medical therapy typically necessitate surgical explantation [14]. In this case, given the absence of intracranial involvement and the superficial nature of infection, an initial conservative approach was reasonable. However, the protracted course highlights the limitations of non-operative management in the setting of suspected biofilm-associated prosthetic infection.

Rationale

Compared with existing literature, this case is notable for the extraordinary duration of drainage, retained cranioplasty material, and repeated exposure to both open surgical and radiosurgical interventions. While case reports exist of post-craniotomy fistula formation, only a few reports describe such prolonged sinus tract persistence in patients with NF2, with most reported lasting months rather than years. The extended timeline illustrates how cumulative surgical trauma, radiation-induced tissue injury, impaired vascularity, and biofilm formation may converge to create a refractory infectious state.

Overall, this case reinforces the importance of recognizing implant-associated biofilm infection in chronic post-craniotomy drainage and supports a multidisciplinary strategy integrating microbiologic optimization, careful radiographic assessment, and timely consideration of surgical intervention when conservative measures fail.

## Conclusions

Repeated surgical trauma, radiation exposure, impaired vascularity, and biofilm formation may predispose patients with neurofibromatosis type 2 to persistent post-craniotomy sinus tract formation. Chronic post-operative drainage unresponsive to antibiotics should prompt evaluation for structural and inflammatory causes. Early imaging, multidisciplinary assessment, individualized management including targeted antimicrobial therapy and definitive surgical intervention when indicated, and long-term surveillance are essential to achieve resolution and reduce long-term morbidity. Further research is needed to better define risk factors and optimize treatment strategies in this complex patient population.
